# Editorial: Research and updates in veterinary transfusion medicine

**DOI:** 10.3389/fvets.2022.1074676

**Published:** 2022-12-07

**Authors:** Shauna L. Blois, Isabelle Goy-Thollot, Eva Spada

**Affiliations:** ^1^Department of Clinical Studies, Ontario Veterinary College, University of Guelph, Guelph, ON, Canada; ^2^University of Lyon, Lyon, France; ^3^Department of Veterinary Medicine and Animal Sciences, University of Milan, Lodi, Italy

**Keywords:** transfusion, blood unit, blood component, blood donor, blood recipient

The knowledge base in veterinary transfusion medicine grows yearly. This issue supports further development in novel aspects of transfusion in veterinary species. Most advancements in veterinary blood transfusion have been focused on domestic species. Transfusion medicine is in its infancy in other species, and this issue presents transfusion practices in novel species.

Evidence-based practices for transfusion are lacking for invertebrates. Gregory, Heniff et al., present a novel serum transfusion technique in the thorny devil stick insect. Gregory, Heniff et al. found that serum, separated from hemolymph collected from donor insects, could be successfully transfused *via* injection into the abdomen of recipient insects, and described potential transfusion reactions in this species. As the authors note, the thorny devil stick insect is a commonly cared for invertebrate in both research and zoological settings. The protocols detailed in this article could help shape future hemolymph transfusion practices in this and other arthropods.

Recently published guidelines regarding pre-transfusion testing in companion animals show the utility of crossmatching in increasing safety of blood transfusion (1). Gregory, Parker et al. explore crossmatching in the development of practical and safe transfusion practices in this endangered pinniped species. While blood types are yet to be defined in the Galapagos sea lion, Gregory, Parker et al. showed that natural antibodies causing transfusion reaction appear to be uncommon in this species. The authors conclude that while pre-transfusion crossmatching is ideal, transfusion could likely proceed safely in an emergency setting when crossmatching is not possible.

Transfusion medicine research in large animals, particularly small ruminants, is a relatively new branch of this discipline. Gastrointestinal parasitism commonly results in anemia in goats. Blood transfusion could be a life-saving procedure in this condition. Finding a donor from the herd, however, can be extremely challenging, as anemia from gastrointestinal parasites is often a herd issue. When a donor of the same species cannot be found, xenotransfusion could be a useful technique. In their study Smith et al. showed that bovine whole blood can be tolerated by goats. Further investigation is necessary to refine the technique for this emergency practice and to better understand the potential adverse effects of xenotransfusion cattle blood to goats.

Whole blood and blood components transfusions are vital treatments in veterinary patients affected by congenital or acquired coagulopaties (3, 4). The study reported by De Pablo-Moreno et al. describes a method for accurately determining and standardizing coagulation Factor V levels as well as coagulation times in *Mus musculus*. The method presented in this research is a contribution to establishing reference intervals and detecting inter-individual differences, and the results obtained are applicable to human or veterinary biomedical research, to transfusion medicine or to pathological models for coagulopathies such as factor V deficiency.

While much of this issue is devoted to introducing transfusion practices in novel species, it also provides information on canine plasma transfusion practices. Component therapy allows for efficient use of blood resources and decreases risk of transfusion reaction by delivering only the blood component(s) needed by a recipient (2). Plasma is commonly stored at −20°C to preserve the activity of coagulation factors and other proteins. Proverbio et al., explore coagulation testing and factor analysis in liquid plasma stored at 2–6°C for up to 1 week. Like others, Proverbio et al. found that coagulation times increased but remained within the reference interval over 7 days of storage (3, 4). Additionally, comparable coagulation factor activity was found in plasma stored for 7 days at 2–6 and −18^o^C, similar to others (4). Never-frozen liquid plasma (Figure 1) provides a quickly available product, avoiding the delays and potential product damage introduced by thawing. Future work can investigate whether never-frozen liquid plasma could provide a suitable alternative to fresh frozen plasma in clinically ill patients, as it does in people.

**Figure 1:**
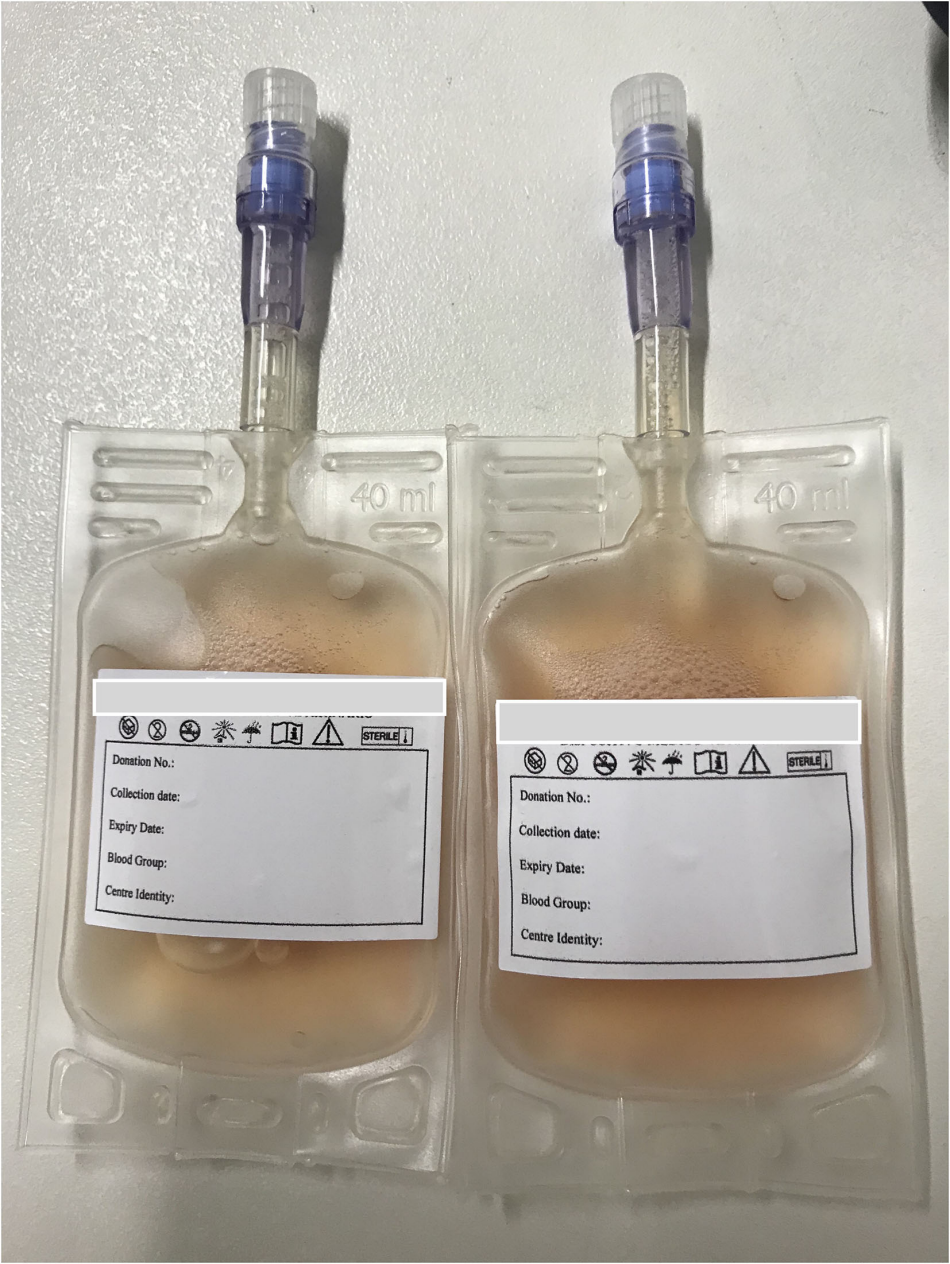
Canine never-frozen liquid plasma ready to use in the volume desired and necessary for the patient's need (property of REVLab, Veterinary Transfusion Research Laboratory, University of Milan, Italy).

Blood transfusion is an increasingly common therapy in veterinary medicine. Safe and practical transfusion therapy is an area of extensive development in companion animals, and an emerging area in novel species. This issue presents novel research to support safe and effective transfusion practices in a spectrum of veterinary patients.

## Author contributions

SB, IG-T, and ES contributed to conception and design of the study. SB wrote the first draft of the manuscript. IG-T and ES wrote sections of the manuscript. All authors contributed to manuscript revision, read, and approved the submitted version.
